# GintAMT3 – a Low-Affinity Ammonium Transporter of the Arbuscular Mycorrhizal *Rhizophagus irregularis*

**DOI:** 10.3389/fpls.2016.00679

**Published:** 2016-05-25

**Authors:** Silvia Calabrese, Jacob Pérez-Tienda, Matthias Ellerbeck, Christine Arnould, Odile Chatagnier, Thomas Boller, Arthur Schüßler, Andreas Brachmann, Daniel Wipf, Nuria Ferrol, Pierre-Emmanuel Courty

**Affiliations:** ^1^Department of Environmental Sciences, Botany, Zurich-Basel Plant Science Center, University of BaselBasel, Switzerland; ^2^Departamento de Microbiología del Suelo y Sistemas Simbióticos, Estación Experimental del Zaidín, Consejo Superior de Investigaciones CientíficasGranada, Spain; ^3^Faculty of Biology, Genetics, Ludwig-Maximilians-University MunichPlanegg-Martinsried, Germany; ^4^Agroécologie, AgroSup Dijon, Centre National de la Recherche Scientifique, Institut National de la Recherche Agronomique, Université Bourgogne Franche-ComtéDijon, France

**Keywords:** arbuscular mycorrhizal fungi, ammonium transporter, low affinity transporter, extraradical mycelium, intraradical mycelium

## Abstract

Nutrient acquisition and transfer are essential steps in the arbuscular mycorrhizal (AM) symbiosis, which is formed by the majority of land plants. Mineral nutrients are taken up by AM fungi from the soil and transferred to the plant partner. Within the cortical plant root cells the fungal hyphae form tree-like structures (arbuscules) where the nutrients are released to the plant-fungal interface, i.e., to the periarbuscular space, before being taken up by the plant. In exchange, the AM fungi receive carbohydrates from the plant host. Besides the well-studied uptake of phosphorus (P), the uptake and transfer of nitrogen (N) plays a crucial role in this mutualistic interaction. In the AM fungus *Rhizophagus irregularis* (formerly called *Glomus intraradices*), two ammonium transporters (AMT) were previously described, namely GintAMT1 and GintAMT2. Here, we report the identification and characterization of a newly identified *R. irregularis* AMT, GintAMT3. Phylogenetic analyses revealed high sequence similarity to previously identified AM fungal AMTs and a clear separation from other fungal AMTs. Topological analysis indicated GintAMT3 to be a membrane bound pore forming protein, and GFP tagging showed it to be highly expressed in the intraradical mycelium of a fully established AM symbiosis. Expression of GintAMT3 in yeast successfully complemented the yeast AMT triple deletion mutant (*MAT*a *ura3 mep1*Δ *mep2*Δ::*LEU2 mep3*Δ::*KanMX2*). GintAMT3 is characterized as a low affinity transport system with an apparent K_m_ of 1.8 mM and a *V*_max_ of 240 nmol^-1^ min^-1^ 10^8^ cells^-1^, which is regulated by substrate concentration and carbon supply.

## Introduction

Nitrogen is an essential, often limiting, macronutrient for plants. Since the availability of nitrogen (N) in form of ammonium (NH_4_^+^) or nitrate (NO_3_^-^) in the environment is quite low, plants have evolved different strategies to overcome this problem. Under natural conditions 70–90% of land plant species are associated with nearly ubiquitous AM fungi, which can increase nutrient and water supply of their host. This goes along with improved plant fitness, growth, and disease resistance. In exchange, the fungal partners receive up to 20% of the photosynthates from the plant (Pearson and Jakobsen, 1993; Graham, 2000; Smith and Read, 2008). Previously it has been assumed that AMF play only a minor role in N nutrition of their host plant. However, several studies testing the contribution of AM fungi to plant N supply revealed that N uptake of the host plant via mycorrhizal uptake pathway can reach 42% (Mäder et al., 2000).

Several studies showed that inorganic NO_3_^-^ and NH_4_^+^ (Bago et al., 1996; Govindarajulu et al., 2005; Jin et al., 2005) or small peptides and amino acids (organic form) (Hawkins et al., 2000) can be absorbed from the soil by extraradical mycelium (ERM) of AMF. There is also some weak evidence that AMF can absorb N from complex organic matter (Leigh et al., 2009; Hodge et al., 2010) and that they take up amino acids from the environment by the expression of amino acid permeases in the ERM (Cappellazzo et al., 2008). Although fungi and plants use many different resources to obtain N, it has been demonstrated that NH_4_^+^ often is the primary N source (Villegas et al., 1996; Hawkins et al., 2000; Toussaint et al., 2004). Assimilation of NH_4_^+^ through the GS/GOGAT pathway is energetically less costly compared to the reduction and assimilation of NO_3_^-^ (Johansen et al., 1996; Marzluf, 1996; Bago et al., 2001; Breuninger et al., 2004; Govindarajulu et al., 2005; Jin et al., 2005).

Once absorbed, most of the inorganic N taken up by the AMF is assimilated and incorporated into arginine, constituting more than 90% of total free amino acids in the ERM. The arginine is translocated to the intraradical mycelium (IRM) (Govindarajulu et al., 2005; Cruz et al., 2007), where it is perhaps bound to the negatively charged polyphosphate in the fungal vacuole, forming a link between nitrogen and phosphorus transport (Martin, 1985; Govindarajulu et al., 2005). In the arbuscule, arginine is metabolized by arginase and urease in the urea cycle, and the free NH_4_^+^ is released into the periarbuscular space where it is taken up by the plant host (Bago et al., 2001; Govindarajulu et al., 2005; Tian et al., 2010).

For a long time it was not clear whether specialized transporters function in the AM symbiotic N exchange. Since the discovery of the first AMTs in *Saccharomyces cerevisiae* (Marini et al., 1994) and *Arabidopsis thaliana* (Ninnemann et al., 1994) several such transporters were characterized in plants (Gazzarrini et al., 1999; Sohlenkamp et al., 2000; Couturier et al., 2007; Guether et al., 2009a), fungi (Javelle et al., 1999, 2003a,b; López-Pedrosa et al., 2006; Lucic et al., 2008; Pérez-Tienda et al., 2011; Ellerbeck et al., 2013) and other organisms (Van Dommelen et al., 1998; Mayer et al., 2006). The so-called high-affinity transporter systems (HATSs) operate in the micromolar range, exhibit saturation kinetics, and the uptake of ammonia leads to depolarization of the transmembrane electrical potential (Ullrich et al., 1984; Wang et al., 1994). In contrast, low-affinity transporter systems (LATSs) are highly active in the millimolar range (Fried et al., 1965; Vale et al., 1988; Wang et al., 1993; Shelden et al., 2001).

Physiological studies in plant roots and the AMF *Rhizophagus irregularis* have revealed that uptake systems for ammonium and nitrate follow biphasic kinetics with respect to external substrate concentrations (Pérez-Tienda et al., 2011). The first AMF AMT, characterized from *R. irregularis* (syn. *Glomus irregularis*, formerly named *Glomus intraradices*), GintAMT1, is a high affinity transporter (López-Pedrosa et al., 2006; Pérez-Tienda et al., 2011). Using immunolocalization and expression analysis of microdissected cells, it was shown that GintAMT1 and a second AMT, GintAMT2 (Pérez-Tienda et al., 2012), were both expressed in the ERM and IRM, participating in the uptake of NH_4_^+^ from the soil solution and possibly in retrieval of NH_4_^+^ leaking out during fungal metabolism at the symbiotic interface. Since then, three related AMTs (GpyrAMT1, GpyrAMT2, GpyrAMT3) were characterized from the glomeromycotan fungus *Geosiphon pyriformis*, which forms a symbiosis with the cyanobacterium *Nostoc* (Ellerbeck et al., 2013).

On the plant side, the expression of several mycorrhiza inducible AMTs could be specifically assigned to arbuscule-colonized cortical cells. Such transporters were identified in *Lotus japonicus* (LjAMT2;2) (Guether et al., 2009b), *Medicago truncatula* (predicted AMT: IMGAG| 1723.m00046) (Gomez et al., 2009), *Glycine max* (GmAMT1;4, GmAMT3;1, GmAMT4;1, and GmAMT4;4) (Kobae et al., 2010), and S*orghum bicolor* (SbAMT3;1, SbAMT4) (Koegel et al., 2013a). The discovery of specialized transporters at the symbiotic interface was an important step to gain more insight into the symbiotic N transfer.

Here we report the discovery, biochemical characterization and localization of GintAMT3, a new AMF AMT from *R. irregularis*, which is expressed primarily in the IRM and represents a low affinity AMT.

## Materials and Methods

### Plant Growth Conditions for Expression Analysis

Experiments were performed with sorghum (*Sorghum bicolor* (L.) Moench), cv Pant-5. This cultivar is closely related to BTx623, the sorghum cultivar used for genome sequencing (Paterson et al., 2009). Seeds of cv Pant-5 were kindly provided by sorghum breeders of I.G.F.R.I. (CCS Agriculture University of Hissar, Haryana, India) and G. B. Pant University of Agriculture and Technology (Pantanagar, Uttaranchal, India). Seeds were surface-sterilized (10 min in 2.5% KClO) and then rinsed with sterile deionized water several times for 1 d and soaked in sterile deionized water overnight. Seeds were pre-germinated on autoclaved sand at 25°C for 24 h and then grown in the dark at room temperature for 72 h. The fungal isolate *Rhizophagus irregularis* BEG-75 (Botanical Institute, Basel, Switzerland) was propagated by trap cultures as previously described (Oehl et al., 2004). To establish AM symbiosis, pregerminated seeds were individually inoculated in compartmented microcosms (Koegel et al., 2013b), where one plant and one hyphal compartment are connected, but separated by two 21 μm nylon meshes and an air gap in between. The air gap was created by placing two 5 mm plastic meshes between the two 21 μm nylon meshes. The two compartments were filled with sterile (120°C, 20 min) growth substrate consisting of a mixture of zeolithe (Symbion, Czech Republic) and sand (1: 1 v/v). About 100 spores were added to the mixture. For the controls (non-mycorrhizal plants), the same amount of autoclaved inoculum was added to the mixture. To correct for possible differences in microbial communities, each pot received 1 ml of filtered washing of AMF inoculum. Plants were grown in a glasshouse with day : night temperatures of c. 28°C : 15°C. Plants were watered twice a week during experiments. From the first week on, 8 ml of modified Hoagland solution was applied weekly. Three different Hoagland solutions, modified after Gamborg and Wetter (1975), were prepared to obtain different N sources or N concentrations : -N, 1x NO_3_^-^ and 1x NH_4_^+^ (Koegel et al., 2013a).

*Populus trichocarpa* (derived from cuttings, clone 10174, Orléans, France) grew together with *S. bicolor*, in a tripartite compartment system, in a zeolithe:sand substrate (1:1; w:w). Thereby, single compartments were separated by 21 μm and 3 mm meshes to allow AMF hyphae but no plant root growth in between the compartments. Plants were inoculated with 1 ml liquid inocula of *R. irregularis*, isolate BEG75 (InoculumPlus, Dijon, France), in 0.01 M citrate buffer (pH 6) containing about 110 spores/ml. Plants were fertilized once a week with 10 ml of Hoagland solution without phosphorus. From the 22nd week on, when all plants showed Pi depletion as indicated by anthocyan accumulation, 10 ml Hoagland solution containing either low Pi ([Pi] = 28 μM) or high Pi ([Pi] = 560 μM) concentration was applied in the compartment for the ERM for 9 weeks. As a control both plant species were grown separately in a single compartment, receiving the fertilizer directly to their root systems.

### *Rhizophagus irregularis* Monoxenic Cultures under Different N Treatments

*Rhizophagus irregularis* monoxenic cultures were established in bi-compartmental Petri dishes to allow separating the root compartment from the hyphal compartment (St-Arnaud et al., 1996; Fortin et al., 2002). Cultures were started on M medium (Chabot et al., 1992) by placing an explant of *Agrobacterium rhizogenes* transformed-carrot (*Daucus carota*) roots colonized with the AMF in the root compartment. Petri dishes were incubated in the dark at 24°C until the hyphal compartment, which contained M medium without sucrose (M–C medium), was profusely colonized by the fungus (~6 weeks). The content of the hyphal compartment was then removed and replaced by liquid M–C medium (15 ml) containing either 3.2 mM NO_3_^-^ (high N) or in a modified M media containing 0.8 mM NO_3_^-^ (low N). The mycelium then colonized this medium over the subsequent 2 weeks. At this point, the medium was removed and replaced by fresh liquid M–C medium without NO_3_^-^. The time of medium exchange was referred as time 0 for the N starvation treatment, and mycelia were harvested 2 and 7 days later. For the N re-supply experiments, mycelia grown in low N media and N-starved for 48 h were supplemented with different N sources and concentrations (3 mM or 30 μM nitrate or ammonium, or 5 mM glutamine) or water (control plates). The ERM was harvested 24 h later. For treatments with acetate or the inhibitor of GS, MSX, the N-starved mycelia (grown in the low N media for 2 weeks and for 2 days in a N-free media) were supplied with 4 mM acetate or 2.5 mM MSX, respectively, together with 3 mM ammonium sulfate. In all experiments, mycelia were collected with forceps, rinsed with sterilized water, dried with sterilized filter paper, immediately frozen in liquid N and stored at -80°C until used. All treatments were independently repeated four times.

### Root Colonization Measurements

A subsample of fresh roots was immersed in 10% KOH and stored in the fridge at 4°C overnight. At the next day the roots were rinsed under the tap and immersed in 2% HCl for 1 h at room temperature. Afterward the roots were rinsed under the tap, immersed in 0.05% trypan blue and stored in the fridge at 4°C overnight. The next day the trypan blue was removed, roots were rinsed with tap water and immersed in lactic-acid glycerol water for destaining. Total root colonization was measured using the grid line intersection method as described by Brundrett et al. (1984). Differences between means of variables were assessed by *t*-test (*p* ≤ 0.5), using Microsoft Excel 2010.

### *In Silico* Analysis

**FIGURE 1:**
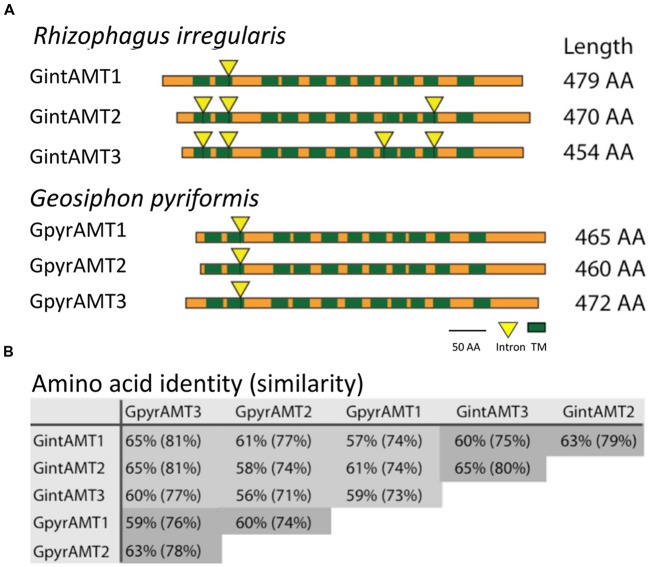
**Topologies of glomeromycotan AMTs and their genetic relationship. (A)** Transmembrane domain (TMD) topology and intron localization of the six glomeromycotan AMTs. Green boxes indicate TMD positions, yellow triangles mark intron positions. Both are highly conserved, while N and C termini differ in length and are less conserved. **(B)** Reciprocal BLAST (Altschul et al., 1997) analysis (Blosum62 matrix) revealed a high conservation at the sequence level between the six transporters. They share at least 56% AA identity and 71% AA similarity. Intra-species comparisons are marked in dark gray.

The sequencing, assembly, and annotation of the *R. irregularis* genome was described in (Tisserant et al., 2012). All *R. irregularis* sequences are available at the Phytozome website^1^ and at GenBank/European Molecular Biology Laboratory (EMBL)/DNA Data Bank of Japan (DDBJ). Using BLAST search and the INTER-PRO domains (IPR018047 and IPR001905) at the JGI website, we identified gene models coding for putative AMTs in the draft genome. Gene prediction at the JGI was performed using gene predictors (FGENESH, and GENEWISE), and gene models were selected by the JGI annotation pipeline (Tisserant et al., 2012). Selection of the AMT models was based on expressed sequence tag (EST) support, completeness, and homology to a curated set of proteins. The putative homologs detected were characterized based on conserved domains, identities, and *e*-values in comparison with fungal AMT sequences available at the NCBI GenBank^2^ and UNIPROT^3^ (**Figure 1**).

Signal peptides were predicted with SignalP 3.0^4^ and subcellular location with TargetP 1.1^5^. Conserved protein domains were analyzed using prosite^6^ and InterProScan^7^.

Full-length amino acid sequences of fungal AMTs were retrieved using BLAST^8^ and the JGI^9^ webpage. Sequence alignments were performed with the ClustalW2 package. For phylogenetic analyses, the alignments were imported into the Molecular Evolutionary Genetics Analyses software (MEGA), version 5.05 (Tamura et al., 2011). Neighbour-joining (NJ) method was applied with the Poisson correction model, the pairwise deletion option and bootstrap test with 1,000 replicates.

A two-dimensional model was generated with Protter – visualize proteoforms (Omasits et al., 2013) and a 3D model was calculated via SWISS-MODEL^10^, based on 2b2hA, an AMT from *Archaeoglobus fulgidus*, AMT-1 (**Supplementary Figure S1**).

### Sampling, RNA Isolation and Quantitative Reverse Transcription-PCR

RNA extraction and cDNA synthesis were performed as described previously (Courty et al., 2009). Primers used as controls or for analysis had an efficiency ranging between 90 and 110%. Plant parts were harvested separately and the ERM was extracted from the substrate by immersing the substrate in water and harvesting the floating mycelium with a 32 μm sieve. Mycelium was snap frozen in liquid nitrogen and stored at -80°C. Plant roots were carefully washed under tap water to remove all soil adhering to the roots. Three subsamples of 100 mg of fresh roots were snap-frozen and stored at -80°C for further gene expression analysis by qRT-PCR.

cDNAs were obtained using the iScript^TM^cDNA Synthesis Kit (BIO RAD Laboratories, Paolo Alto, CA, US). For quantification a two-step quantitative RT-PCR (qRT-PCR) approach was used. Gene specific primers were designed in Primer 3^11^ and amplify 3.1^12^. Target gene expression was normalized to the expression of the transcription elongation factor TEF1α in *R. irregularis.* qRT-PCRs were run in a 7500 real-time PCR systems (Applied Biosystems) using the following settings: 95°C for 3 min and then 40 cycles of 95°C for 30 s, 60°C for 1 min and 72°C for 30 s. For each transporter three biological and three technical replicates (*n* = 9) per treatment were conducted.

### Isolation of GintAMT3 and Functional Expression in Yeast

Full-length doubled-stranded cDNA was synthesized from RNA of the ERM using the SMARTer^TM^ cDNA Synthesis Kit (Clontech, US, Canada). GintAMT3 (JGI Protein ID: 218175; JGI Transcript ID: 218287; NCBI accession number: KU933909) was then amplified using the primer pair GintAMT3_fl_Fwd/GintAMT3_fl_Rev (**Supplementary Table S1**). Full-length GintAMT3 was cloned into pDR196 using the Gateway technology (Invitrogen), as described previously (Wipf et al., 2003), resulting in the pDR196-GintAMT3 plasmid construct. pDR196-GintAMT3 and as a control the empty vector were transformed into the *Saccharomyces cerevisae* strain 31019b (*MAT*a *ura3 mep1*Δ *mep2*Δ::*LEU2 mep3*Δ::*KanMX2*) (Marini et al., 1997) as described by Dohmen et al. (1991). Transformants were selected on SD media lacking uracil and further transferred on yeast nitrogen base (YNB-N) glucose media without ammonium and amino acids supplemented with NH_4_Cl as the sole nitrogen source (1 and 3 mM). Sequence identities and integrities were verified by sequencing.

### [^14^C]Methylamine Uptake Assay

Initial [^14^C]methylamine uptake rates (American Radiolabeled Chemicals, Inc., St. Louis, MO, USA) for amino acids were measured as described previously (Marini et al., 1997). Single colonies were grown in liquid YNB-N supplemented with 6% glucose and 500 μg/mL L-proline to logarithmic phase and were centrifuged at an OD_600_ of 0.5 to 0.8. Cells were washed twice in sterile water and resuspended in 50 mM KH_2_PO_4_ buffer pH 5, to a final OD_600_ of 5. Before the uptake measurements an aliquot of yeast cells was supplemented with 20 mM glucose, incubated at 30°C for 5 min at 1,000 rpm. To start the reaction an equal amount of pre-warmed KH_2_PO_4_ buffer containing 15 kBq of [^14^C]methylamine and unlabelled methylamine (0–15 mM) was added. Cells were incubated at 30°C, 1,000 rpm, and 45 μl subsamples were taken after 1, 2, 3, and 4 min, diluted in 5 ml KH_2_PO_4_/sorbitol buffer, separated from the incubation buffer on glass fibre filters (Whatman), and washed twice with the same buffer. Radioactivity retained on the filter was assayed by liquid scintillation spectrometry (Packard).

### Expression Analysis at the Cellular Level by Laser Capture Microdissection

*Sorghum* roots were washed with tap water to remove the substrate. Pieces of 10–15 mm were cut with a razor blade from differentiated regions of the mycorrhizal and non-mycorrhizal roots. The root segments were embedded in OCT (EMS, Delta Microscopies Aygues-Vives, France) and then frozen at -23°C. 40 μm thin sections were cut with a Cryocut (Cryocut 1800 Leica), and the cuts were placed on Fisher Probe-On slides (Fisher Scientific, Ilkirch, France). The sections were washed and fixed as follows: 3 min 70% EtOH, 30 min DEPC H_2_O, 2 min 100% EtOH. The slides were then dried for 20 min at 37°C on a warming plate and kept at -80°C before use.

An Arcturus XT microdissection system (Applied Biosystems, Foster City, CA, USA) was used to collect the cells from the mycorrhizal and non-mycorrhizal root sections. Eight replicates of two different cell types were collected: arbuscule-containing cells (ARBs), and cortical cells from non-mycorrhizal roots (Cs). A total of 5,000–15,000 cells were cut out for each sample. RNA from collected cells was extracted using the Arcturus Pico Pure RNA isolation Kit (Excilone, Applied Biosystems, Foster City, CA, USA), with an in-column DNase treatment following manufacturer’s instructions. Quantity and quality of the extracted RNAs were verified using a bioanalyzer with RNA pico chips (Agilent, Santa Clara, CA, USA). Synthesis of cDNA and quantitative reverse transcriptase polymerase chain reaction (qRT-PCR) analysis was done as previously described using the iScript cDNA Synthesis kit (Bio-Rad, Hercules, CA, USA), starting with 100 pg RNA.

## Results

### *In Silico* Analysis of GintAMT3

Based on the high conservation of amino acid sequences, a consensus signature for AMTs has been defined corresponding to Prosite PDOC00937, InterPro IPR001905, and Pfam 00909. The *ab initio* annotation and subsequent automated BLAST and INTERPRO searches of the *R. irregularis* draft genome sequence (Tisserant et al., 2012) identified three gene models containing these conserved AMT domains, from which two were already characterized, namely *GintAMT1* (López-Pedrosa et al., 2006) and *GintAMT2* (Pérez-Tienda et al. (2012). The length of the nucleotide sequence of *GintAMT3* is 1,798 bp. The coding exon sequence (1,365 bp) was confirmed by EST alignment and cDNA sequencing, and it is interrupted by four short introns of 92 bp, 130 bp, 125 bp, and 86 bp length, typical of *R. irregularis* (Tisserant et al., 2012).

Comparisons between cDNA and genomic sequences of the *R. irregularis* AMT genes revealed 1, 3, and 4 introns for *GintAMT1*, *GintAMT2*, and *GintAMT3*, respectively. Their positions are conserved between the genes, whenever present in more than one gene (**Figure 1A**). Location of intron 2 is even conserved in all six AMT genes of *R. irregularis* and *G. pyriformis*, indicating its presence in a common ancestral gene before these glomeromycotan species split. This is remarkable as the two AMF are distantly related and probably have separated more than 400 million years ago (Schüßler et al., 2001). A comparison with an AMT gene of the basidiomycete *Ustilago maydis*, *UmUMP2*, revealed an intron in a different position, 60 base pairs further downstream between codons for two other highly conserved residues, a glycine and an asparagine residue. Thus, the position of intron number 2 is conserved among glomeromycotan AMT genes but, based on present data, also appears to be specific for this phylum. The 6 encoded proteins show high levels of amino acid identity and similarity (**Figure 1B**).

The introns 1 and 4 are conserved between *GintAMT2* and *GintAMT3*, suggesting recent gene duplication. Intron 3 only exists in *GintAMT3*. Also the positions of predicted transmembrane domains (TMDs, green rectangles in **Figure 1A**) are highly conserved between the AMF AMTs.

**FIGURE 2:**
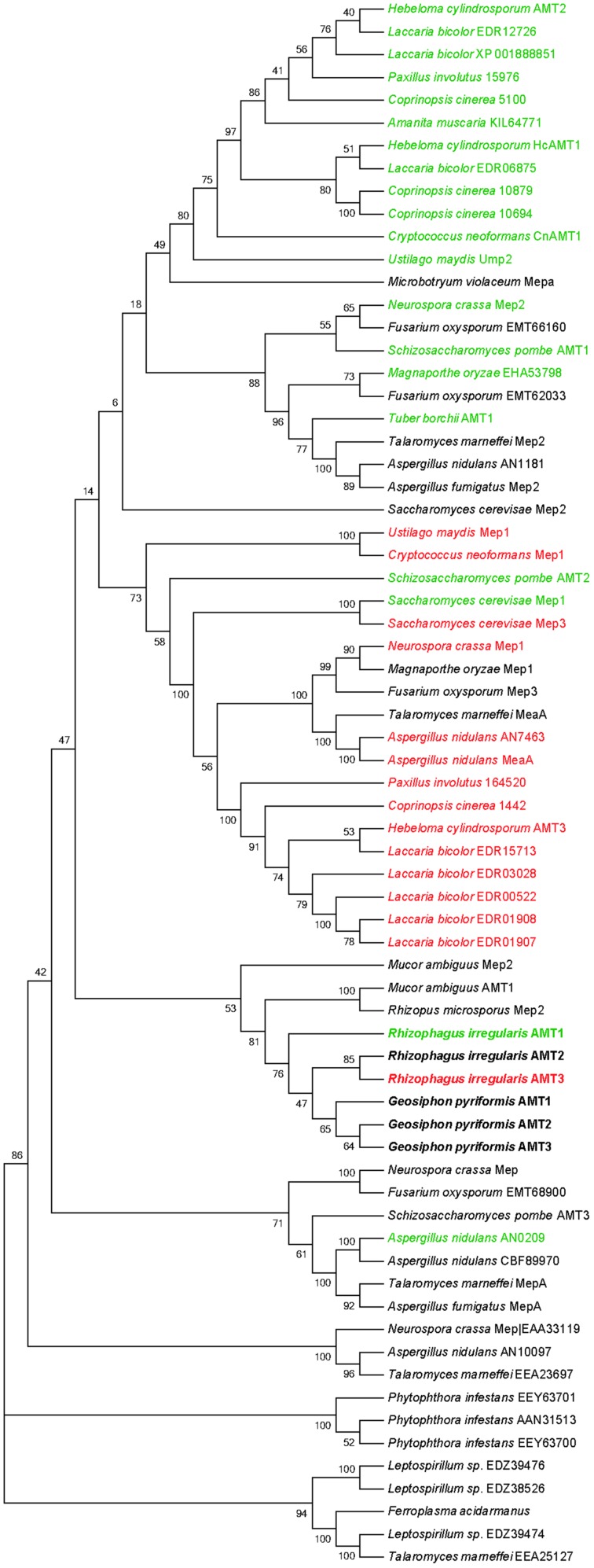
**Phylogenetic tree (“NJ Bootstrap Consensus Tree”) of fungal Mep/AMT proteins.** Transporter names or accession numbers are indicated. Bootstrap values are derived from 1000 replications. HATSs are highlighted in green and low affinity transporter are highlighted in red. Glomeromycotan AMTs are highlighted in bold. Phylogenetic tree was constructed using MEGA6.06 package (Tamura et al., 2013). Gene names, protein IDs and bootstapping values are indicated. Sequences obtained from the JGI databank: *Aspergillus nidulans* AMT (AN7463), AMT (AN0209), AMT (AN10097), AMT (AN1181); *Coprinopsis cinerea* AMT (1442), AMT (5100), AMT (10879), AMT (10694); *Rhizophagus irregularis* AMT1 (337025), AMT2 (314209), AMT3 (21817); *Paxillus involutus* AMT (164520), AMT (15976), AMT (KIJ11108). Sequences obtained from the *NCBI* databank*: Aspergillus fumigatus* Mep2 (EAL90420), MepA (EAL91508); *Amanita muscaria* (KIL64771); *Cryptococcus neoformans* Mep1 (XP_566614), AMT1 (XP_567361); *Ferroplasma acidarmanus* (WP_019841313); *Fusarium oxysporum* AMT (EMT62033), AMT (EMT68900), AMT (EMT66160), Mep3 (EMT61925); *Geosiphon pyriformis* AMT1 (AGO45860), AMT2 (AGO45861), AMT3 (AGO45862); *Hebeloma cylindrosporum* AMT1 (AAM21926), AMT2 (AAK82416), AMT (AAK82417); *Laccaria bicolor* AMT (EDR12726), AMT (EDR06875), AMT (EDR03028), AMT (EDR01908), AMT (EDR01907), AMT (EDR00522), AMT (EDR15713), AMT (XP_001888851); *Leptospirillum sp.* AMT (EDZ39474), AMT (EDZ39476), AMT (EDZ38526); *Magnaporthe oryzae* AMT (EHA53798), Mep1 (EHA48931); *Microbotryum violaceum Mepa* (AAD40955); *Neurospora crassa* Mep1 (EAA35174), Mep2 (EAA32441), Mep (KHE86570), Mep (EAA33119); *Mucor ambiguous* AMT1 (GAN10886), Mep2 (GAN10300); *Phytophthora infestans* AMT (AAN31513), AMT (EEY53846), AMT (EEY63701), AMT (EEY63700); *Rhizopus microspores putative* Mep2 (CEJ04454); *Saccharomyces cerevisae* Mep1 (P40260), Mep2 (P41948), Mep3 (P53390); *Schizosacchromyces pombe* AMT1 (NP_588424), AMT2 (CAB65815), AMT3 (P53390); *Talaromyces marneffei* MepA (EEA28528), MeaA (EEA28073), Mep2 (EEA20421), putative AMT (EEA25127), putative AMT (EEA23697); *Tuber borchii* AMT1 (AAL11032); *Ustilago maydis* Mep1 (KIS67424), UMP2 (KIS66151).

A phylogenetic analysis was performed to compare the protein sequences of the glomeromycotan AMTs with the ones from other fungi. This analysis revealed a close relationship of the six glomeromycotan AMTs, and a clear homology with one AMT family of the Ascomycetes, represented by SpAMT1 (**Figure 2**). For the non-glomeromycotan AMTs, we observed a clear separation of the AMTs according to their affinities, with the exception of the *S. cerevisiae* high-affinity transporter ScMEP2, which is more closely related to the low affinity *S. cerevisiae* AMTs than to its orthologs in other fungi (**Figure 2**).

### Root Colonization Depending on N and P Conditions

After 30 weeks of growth, symbioses between *R. irregularis* and the two host plants, poplar and sorghum, were well established (**Supplementary Table S2**). Root hyphal colonization rates ranged between 79 and 93% and were not significantly different (*n* = 7). In sorghum, three times more arbuscules were found in the low Pi treatment as compared to the high Pi treatment, while no significant differences were found in poplar. In the 3 months old sorghum plants, set under three different N conditions, hyphal colonization ranged between 94 and 99% (*n* = 4) (**Supplementary Table S2**).

### Yeast Complementation, GFP Localization, and Ammonium Uptake

**FIGURE 3:**
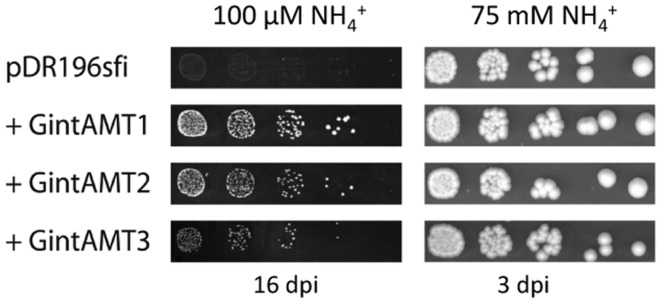
**Complementation of ammonium uptake deficiency in a yeast triple mutant by glomeromycotan AMTs.** Ammonium uptake-deficient yeast cells (*mep1–3*Δ) were transformed with an expression vector containing various AMT genes under control of the strong *PMA1* promoter. Fivefold dilution series of the transformants were incubated either on minimal medium containing 50 μM (NH_4_)_2_SO_4_ (=100 μM NH_4_^+^) as sole nitrogen source for 16 days **(left)** or on synthetic complete medium [containing roughly 37.5 mM (NH_4_)_2_SO_4_] for 3 days **(right)**. *Rhizophagus irregularis* AMTs are able to partly complement the growth deficiency of the Δ*mep1–3* yeast mutant on low NH_4_^+^ concentrations (100 μM, **left**).

The putative transporter gene *GintAMT3* was tested for complementation of the yeast *mep1-3*Δ mutant (strain MLY131a/α, Lorenz and Heitman, 1998) in comparison with the already known AMT genes. Cells were transformed with variants of the plasmid pDR196*sfi* containing the different AMT genes or a stuffer gene (a part of a human aldolase gene without ORF) cloned into the *SfiI* sites. The genes were constitutively expressed under the *PMA1* promoter. All three transporter genes of *R. irregularis* at least partly restored the ammonium uptake capability in yeast, as proven by their capability to restore growth of the *mep1-3*Δ mutant on medium containing 50 μM (NH_4_)_2_SO_4_ as sole nitrogen source (**Figure 3**). *GintAMT1* complemented more efficiently the mutant phenotype than GintAMT2 and GintAMT3, demonstrated by larger colonies in a successive 5x dilution series on medium containing 50 μM (NH_4_)_2_SO_4_ as sole nitrogen source (**Figure 3**).

**FIGURE 4:**
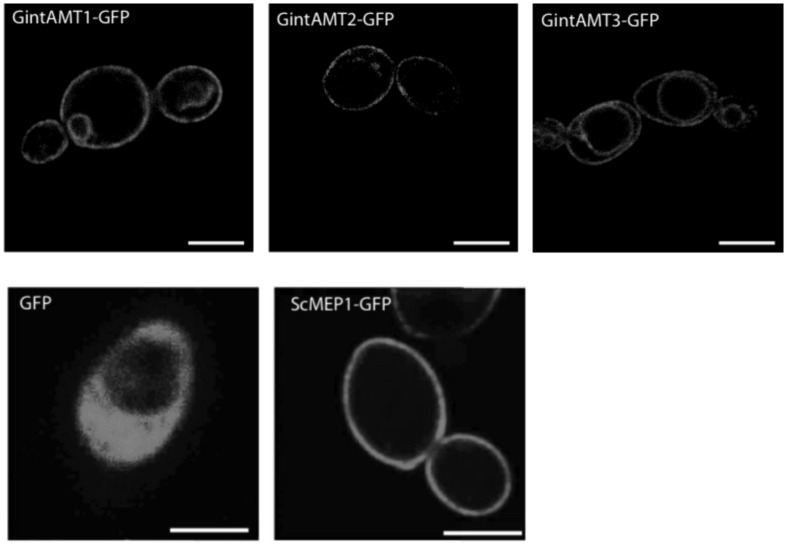
**Localization of GFP-tagged glomeromycotan AMTs in *S. cerevisiae.*** C-terminally GFP-tagged versions of ScMEP1, GintAMT1, GintAMT2, and GintAMT3 as well as soluble GFP were cloned into pDR196sfi and expressed in *S. cerevisiae* under control of the *PMA1* promoter. The cells were grown to logarithmic growth phase and the localization of the fusion proteins was assessed by confocal microscopy. All three *R. irregularis* AMTs tagged with GFP were localized at the plasma membrane (PM), like ScMEP1-GFP (**lower center**), the positive control. Soluble GFP was localized to the cytoplasm of *S. cerevisiae* (**lower left**). Additional vacuolar membrane localization was visible for GintAMT3-GFP (**upper right**). GintAMT1-GFP (**upper left**) displayed an additional nuclear membrane localization. Bars are 2.5 μm.

To test if the different complementation efficiencies observed by the different AMTs could be due to an incorrect protein localization in the heterologous system, we cloned *GintAMT1*, *GintAMT2*, and *GintAMT3* to the 5′ end of a green fluorescent protein (GFP) coding gene into the expression vector pDR196*sfi* and transformed the yeast *mep1–3*Δ mutant with these constructs, resulting in the expression of C-terminal GFP-tagged AMT fusion proteins in yeast cells. The localization of these fusion proteins was performed with a Leica SP5 confocal laser-scanning microscope (CLSM, **Figure 4**). All tagged proteins were localized to the plasma membrane (PM) in *S. cerevisiae* (**Figure 4**). Additionally, we observed vacuolar or perinuclear membrane localization for some of them indicative of an endoplasmic reticulum localization, most probably as an overexpression artifact (**Figure 4**). All tagged transporters behaved like the untagged versions (not shown), either complementing the growth defect of the yeast mutant (GintAMT1-GFP, GintAMT2-GFP, GintAMT3-GFP) or not (soluble GFP).

### Ammonium Removal Assay

**FIGURE 5:**
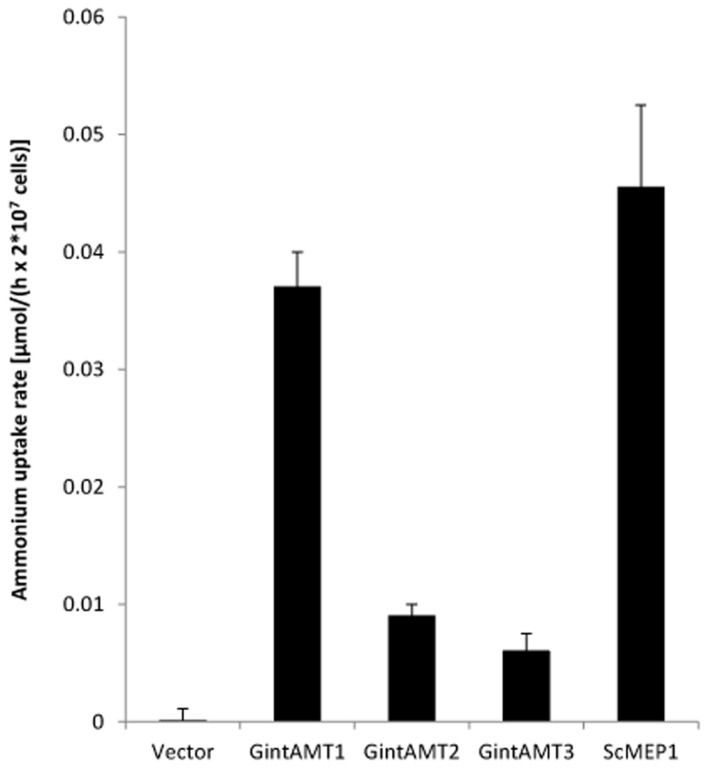
**Quantification of ammonium uptake in yeast cells expressing glomeromycotan AMTs.** Yeast cells expressing the stated genes from the plasmid pDR196sfi were grown over night in synthetic complete medium lacking uracil (HC-U), washed and cultured in liquid medium containing a starting concentration of 2 mM ammonium. Samples were taken after 10, 30, 60, 120, 180, 240, and 300 min, and residual ammonium was determined. Yeast cells expressing *ScMEP1* and *GintAMT1* took up ammonium quite rapidly. *GintAMT2* and *GintAMT3* expressing cells imported ammonium at a slower rate, but clearly above background level (“Vector”). Bars show average of 3–4 experiments and standard deviation.

To measure the different ammonium transport capacities of the transporters, ammonium removal assays according to Ellerbeck et al. (2013) were performed. In this experimental setup, dense yeast cultures (OD_600_ = 2) were incubated in relatively high ammonium concentrations (1 mM) for several hours and the remaining ammonium in the medium was measured at distinct time points (after 10, 30, 60, 120, 180, 240, and 300 min). Therefore, no kinetics but overall ammonium uptake can be measured. The results of the removal assays confirmed the yeast complementation assays. The 3 AMTs of *R. irregularis* transported ammonium to a varying but always lower extent than ScMEP1 (**Figure 5**). GintAMT2 and GintAMT3 showed lower ammonium removal activity in these experiments (**Figure 5**) than GintAMT1, supporting the results from the complementation assays on plate (**Figure 3**).

### *GintAMT* Expression Levels

**FIGURE 6:**
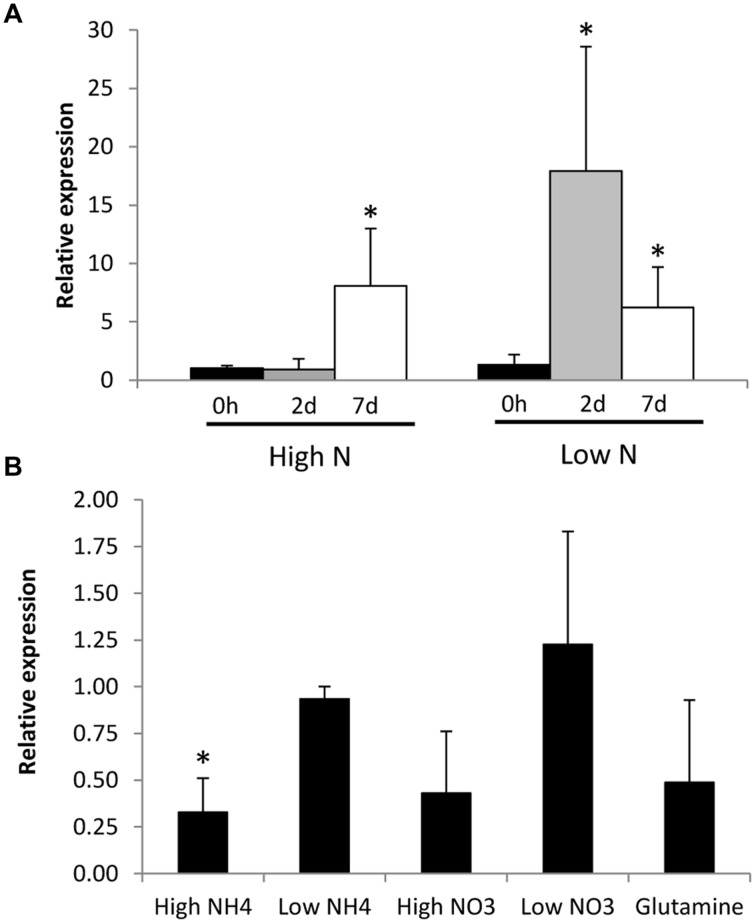
**Effect of N availability on *Gint*AMT3 gene expression. (A)** Real-time RT-PCR analysis of *GintAMT3* mRNA levels in the ERM of *R. irregularis* developed in liquid M-C medium in the presence of either 3.2 mM (High N) or 0.8 mM nitrate (Low N) and exposed for different periods of time to a N-free medium. **(B)** Effect of N addition to the N-starved mycelium on *GintAMT3* expression. Gene expression was analyzed by real-time RT-PCR in ERM grown in 25% N media, maintained for 48 h in a N-free media (Control) and exposed for 24 h to 3 mM (High) or 30 μM (Low) of NH_4_^+^ or NO_3_^-^, or 5 mM glutamine. Control plates were supplemented with H_2_O. Data were calibrated by the expression values obtained for the gene encoding the EF1α. Error bars represent SE of the mean of three independent experiments. ^∗^: statistically significant (*p* < 0.05) in comparison to the respective control value.

Regulation of *GintAMT3* gene expression by N starvation was assessed in the ERM of *R. irregularis* developed in monoxenic cultures in M–C medium (standard or high N) or in a modified medium containing reduced N (low N), and then incubated for different periods of time in a N-free M medium. *GintAMT3* transcript levels increased when the fungus was exposed to the N-free medium. When the fungus was grown in the low-N media, *GintAMT3* up-regulation was observed 2 days after N deprivation, while in the ERM grown in the high N medium *GintAMT3* up-regulation was observed 5 days later (**Figure 6A**).

To further investigate the effect of N on *GintAMT3* transcript levels, we also determined whether the addition of different N sources to the N-deprived mycelia had an effect on its expression (**Figure 6B**). Relative to the N-deprived ERM, *GintAMT3* transcript levels significantly decreased 24 h after the addition of 3 mM NH_4_^+^. Feeding the mycelium with nitrate, glutamine, or 30 μM NH_4_^+^ did not significantly change *GintAMT3* gene expression, although a slight decrease was observed after the addition of 3 mM nitrate or glutamine.

**FIGURE 7:**
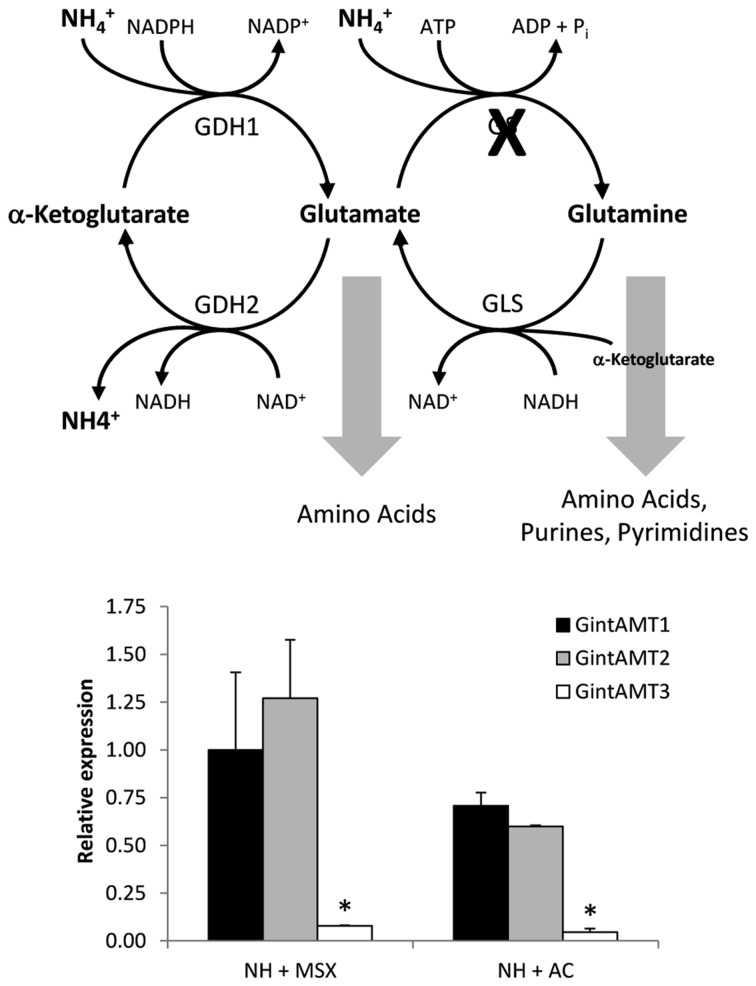
**Effect of MSX and acetate on *GintAMTs* gene expression.** Gene expression was analyzed by real time RT-PCR in the N-deprived mycelia (NH) and supplied for 24 h to 3 mM NH_4_^+^ plus 2.5 mM MSX (NH + MSX), or 3 mM NH_4_^+^ plus 4 mM acetate (NH + AC). Data were calibrated by the expression values obtained for the gene encoding the EF1α. Error bars represent SE of the mean of three independent experiments. ^∗^: statistically significant (*p* < 0.05) in comparison to the respective control value.

The effect of the GS inhibitor MSX on the expression levels of the three *R. irregularis* AMT genes was also tested. For this purpose, the N-deprived ERM was incubated for 24 h in the presence of 2.5 mM MSX in the NH_4_^+^ re-supplementation media. Under these conditions, NH_4_^+^ should be accumulated and glutamine should be depleted. MSX caused a down-regulation of *GintAMT3* gene expression, but did not have any effect on *GintAMT1* and *GintAMT2* transcript levels (**Figure 7**). To determine if transcription of the *R. irregularis* AMT genes were affected by carbon supply, *GintAMT*s gene expression was assessed in the N-deprived ERM supplemented with NH_4_^+^ and acetate, a carbon source taken up and assimilated by the ERM (Pfeffer et al., 1999). Relative to the N-deprived mycelium, supplying the ERM with ammonium and acetate induced down-regulation of the three *GintAMT*s, with the strongest and statistically significant effect for *GintAMT3* (**Figure 7**).

**FIGURE 8:**
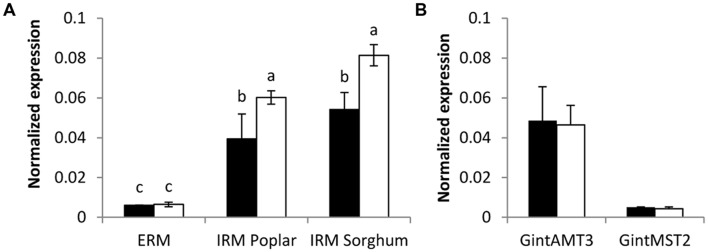
**Quantification of *GintAMT3* under phosphate stress.** Gene expression was measured by quantitative polymerase chain reaction in the ERM and IRM of **(A)** inoculated *P. trichocarpa* and *S. bicolor* and in **(B)** microdissected arbusculated cells in *S. bicolor*. **(A)** The sorghum and poplar plants grew in a tripartite compartment system where only the fungus had access to the high phosphorous source (open bars) or low phosphorous source (closed bars). Differences between ERM and IRM were tested with a one-way ANOVA. Data were calibrated by the expression values obtained for the gene encoding the transcription elongation factor TEF1α. Values are means of nine replicates, error bars represent SD. Difference between treatments were tested with a one-way ANOVA. Lower case letters indicate significant difference (Tukey’s *t*-test; *p* < 0.05). **(B)** Inoculated *S. bicolor* grew in a two-partite compartment system where only the fungus had access to the high phosphorous (open bars) or low phosphorous (closed bars) source. Arbusculated cells were laser microdissected and transcript abundances of *GintAMT3* and *GintMST2* (monosaccharide transporter essential for functional symbiosis, Helber et al. (2011)) as a positive control were measured by qPCR. Data were calibrated by the expression values obtained for the gene encoding the transcription elongation factor TEF1α. Values are means of six replicates, error bars represent SD. Difference between treatments was tested with Tukey’s *t*-test (*p* < 0.05).

**FIGURE 9:**
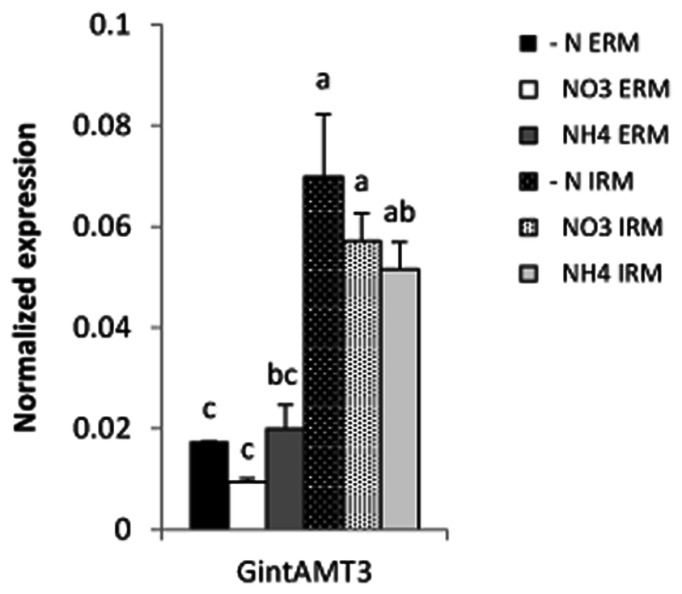
**Quantification of *GintAMT3* expression in *R. irregularis* by qPCR.** Inoculated *S. bicolor* grew in a two-partite compartment system where only the fungus had access to the second compartment. In this system only the fungus had access to the applied nutrients. Hyphal compartments received either Hoagland solution containing no nitrogen source (–N), or nitrate (NO_3_) or ammonium (NH_4_) as the sole nitrogen source. Gene expression of *GintAMT3* was measured in the ERM and IRM. Data were calibrated by the expression values obtained for the gene encoding the transcription elongation factor TEF1α. Values are means of nine replicates, error bars represent SD. Difference between treatments were tested with a one-way ANOVA. Lower case letters indicate significant difference (Tukey’s *t*-test; *p* < 0.05).

Expression of all three *R. irregularis* AMT was assessed in ERM and IRM when the fungus was associated with poplar and sorghum. In this experimental set-up the fungus had either access to a low Pi source or a high Pi source. The expression level for the high affinity transporter GintAMT1 was low and similar in the ERM and in the IRM, independently of the Pi availability. GintAMT2 was strongly expressed in the ERM and IRM, independently of Pi availability (**Supplementary Figure S2**). Expression level of *GintAMT3* was far higher in the IRM than in the ERM. *GintAMT3* was significantly more strongly expressed under high Pi conditions compared to low-Pi in the IRM (**Figure 8A**). Expression patterns of all three transporters were the same in both plant species. When we measured gene expression of *GintAMT3* in laser-microdissected arbusculated cells we did not observe significant differences between high Pi and low Pi condition (**Figure 8B**). Moreover, *GintAMT3* expression was at least twice as high in the IRM as compared to the ERM, independent of the N source (**Figure 9**).

### [^14^C]Methylamine Uptake Assay

**FIGURE 10:**
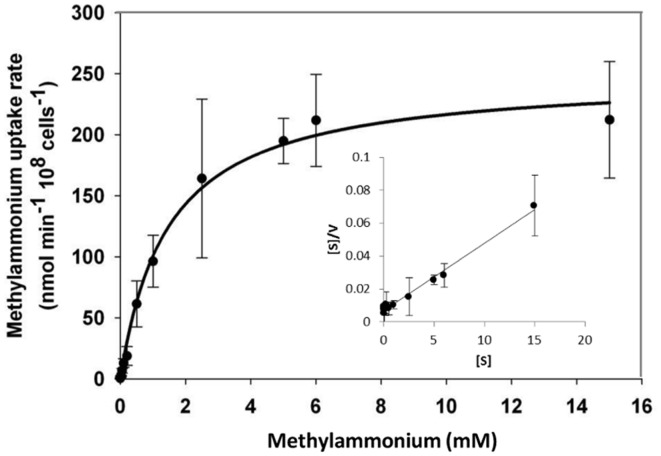
**Biochemical characterization of GintAMT3.** Heterologous expression of pDR196-GintAMT3 in yeast strain 31019b (*MAT*a *ura3 mep1*Δ *mep2*Δ::*LEU2 mep3*Δ::*KanMX2*) (Marini et al., 1997). [^14^C]methylamine uptake rates were measured at pH 5 at different substrate concentrations. Inset: Hanes–Woolf plot.

Functional expression of *GintAMT3* in the yeast triple mutant revealed it to be a low affinity transporter with an apparent K_m_ of 1.8 mM and a V_max_ of 240 nmol^-1^ min ^-1^ 10^8^ cells^-1^. We observed a steep increase in methylamine uptake until reaching a plateau at about 6 mM. However, increasing the amount of supplied methylamine showed that GintAMT3 is still able to take up methylamine at a steady pace (**Figure 10**).

## Discussion

In the AM symbiosis, the main role of the AM fungal partners is the acquisition of mineral nutrients from the soil, in the ERM, and the transfer of these nutrients to the IRM and from there, by way of the periarbuscular space, to the plant. Though P is the most-often named mineral nutrient in this context, N can be a limiting factor for plant growth as well, and the N delivered by AMFs may play an important role for plant growth and health. According to current knowledge, AMF take up N in the ERM, preferentially in form of ammonium, metabolize it to arginine in the GS/GOGAT pathway and in the urea cycle, and transport it to the IRM in the form of arginine (Casieri et al., 2013). At the plant fungal interface (in the arbuscule), ammonia is thought to be released from arginine through the action of arginase and urease and then transported to the plant. For the plant partner, it has been shown already that the expression of certain AMTs is specifically upregulated in arbuscule-containing cells, and that that these plant AMTs reside in the periarbuscular membrane. However, not much is known yet about the localization and regulation of the fungal AMTs involved in this process. In our study, we describe a new functional AMT, GintAMT3, of *R. irregularis*, and we try to characterize its role in the symbiotic N transfer.

### AMF Ammonium Transporters: A Separated Phylogenetic Group

Sequence homology analysis revealed high intraspecific and interspecific sequence conservation of GintAMT3 to the two already known AMTs of *R. irregularis* (López-Pedrosa et al., 2006; Pérez-Tienda et al., 2011) and the three AMTs previously identified in *Geosiphon pyriformis* (Ellerbeck et al., 2013) (**Figure 1**). All six glomeromycotan AMTs shared high sequence similarity and the 11 TM helices of AMTs. Positioning of the intron sequences showed further, that the glomeromycotan AMT genes are highly conserved. The closest homolog to GintAMT3 is GintAMT2, which shared 80% of sequence similarity. The additional intron sequences in the GintAMT2 and GintAMT3 genes suggested a recent gene duplication event.

Phylogenetic analysis of AMTs from Ascomycota, Basidiomycota, Zygomycota and Glomeromycota revealed that the six AMTs from Glomeromycota species (three from *R. irregularis*) clustered separately from the HATS and LATS of Ascomycota and Basidiomycota (**Figure 2**), indicating a distinct AMT evolution in these fungal phyla. Note that some of the AMTs of Ascomycota and Basidiomycota have been identified in species forming ectomycorrhizas, such as *Tuber borchii* (Montanini et al., 2002), *Hebeloma cylindrosporum* (Javelle et al., 2001, 2003a,b), *Amanita muscaria* (Willmann et al., 2007), and *Laccaria bicolor* (Lucic et al., 2008).

### GintAMT3 Is a Low Affinity Transporter System

Both a LATS and a HATS have already been described in *R. irregularis* (Pérez-Tienda et al., 2012). GintAMT1 has been characterized as a HATS with an apparent K_m_ of 26 μM (López-Pedrosa et al., 2006). The kinetics of the second AMT, GintAMT2, could not be determined by methylammonium uptake assay (Pérez-Tienda et al., 2011), but qRT-PCR measurements revealed that GintAMT2 is constitutively expressed under N-limiting conditions, suggesting a role in ammonium retention rather than in ammonium uptake (Pérez-Tienda et al., 2011). We characterized GintAMT3 as a LATS with an apparent K_m_ of 1.8 mM and a V_max_ of 240 nmol^-1^ min^-1^ 10^8^ cells^-1^. In our experiments, expression of GintAMT3 is dependent on the N nutritional status of the AM fungus but independent from the provided N source under N limiting conditions. Severe N stress induced expression of GintAMT3 independently of the supplied N source and abundance of GintAMT3 transcript decreased within a few days. These results indicate the existence of unknown regulatory mechanisms involved in transcriptional or post-transcriptional regulation of AMTs in AMF. Further, we could show that GintAMT3 expression is not only dependent on N nutrition status but also on fungal carbon status, indicating a tight connection to symbiotic interactions. A similar observation was reported in *Hebeloma cylindrosporum*, a Basidiomycota fungus forming ectomycorrhizal symbiosis (Javelle et al., 2003b).

Using a compartmented system, we analyzed fungal nutrient transporterts in the ERM and IRM when associated with *Sorghum bicolor*. Independently of the N source, the expression level of GintAMT3 in the IRM was significantly more than twofold induced compared to the ERM. As P is also a major nutrient transferred by the AM fungus to the plant, we assessed the effect of P availability on GintAMT3 expression in the ERM and IRM when *R. irregularis* was associated with *S. bicolor* or with poplar, and found an induction of GintAMT3 in the IRM by P. The high expression of GintAMT3 in the IRM indicates that it might be located in the arbuscules.

Microdissection of *S. bicolor* roos revealed indeed that GintAMT3 is expressed in the symbiotic root tissue, and specifically in arbuscule-containing cells. Heterologous expression of GFP tagged GintAMT3 in yeast revealed localization of the AMT in the PM and vacuolar membrane. Given that current experimental evidence supports a role for AMT proteins in ammonium uptake (Khademi et al., 2004; Lamoureux et al., 2010) and that ammonium is the N form taken up by the plant at the arbuscular interface (Govindarajulu et al., 2005; Tian et al., 2010), expression of AMT genes in the arbuscules indicates that there might exist a competition between the plant and the fungus for N that is present in the interfacial apoplast (Guether et al., 2009a). As it was proposed for the high-affinity transporters GintAMT1 and GintAMT2, the high expression of GintAMT3 in the arbuscules also suggests a role for this transporter in ammonium retrieval from the periarbuscular space, but in situations where the ammonium concentrations are high. Additionally to its incorporation in metabolism, the vacuolar localization of GintAMT3 indicated that ammonium could be stored in vacuoles to maintain low cytoplasmic ammonium concentrations as shown in yeast (Soupene et al., 2001) or plants (von Wirén et al., 2000; Loqué et al., 2005), or in intracellular vesicles (Chalot et al., 2006). Studies on ammonium/methylammonium transporters (AMT/MEP) of enteric bacteria have shown that these transporters function as ammonia channels. Ammonium is deprotonated at the channel entrance and ammonia is transported through it. The transport through the channel is energy-independent and bidirectional (Soupene et al., 1998, 2002; Khademi et al., 2004). Therefore, it might also be possible that GintAMT3 function as a bidirectional transporter for import and export of ammonium from the vacuole. Furthermore, it is also possible that GintAMT3 functions as an export carrier for ammonium from the arbuscules to the periarbuscular space. However, to assess possible bidirectional transport properties of GintAMT3, patch clamp measurements are necessary. Knockdown of GintAMT3 by host induced gene silencing and virus induced genes silencing could illustrate the importance of this transporter for a functional symbiosis (Helber et al., 2011).

## Conclusion

Here, we demonstrate that GintAMT3 encodes a functional low affinity transporter. We show that it is localized in the fungal membrane, and that it is expressed in the ERM and IRM of colonized poplar and sorghum plants. Increased expression in the IRM under high-P conditions indicates further that more ammonium is transferred when the AM fungus has increased access to a P source.

## Author Contributions

SC, JP, and ME made the *in silico* analysis. ME, SC, and OC made the yeast complementation. SC and OC performed the methylamine uptake assay. SC and CA performed laser capture microdissection. CA and ME made the GFP localization. SC and JP made root colonization and expression analysis. JP performed MSX and acetate assays. All co-authors participate in writing.

## Conflict of Interest Statement

The authors declare that the research was conducted in the absence of any commercial or financial relationships that could be construed as a potential conflict of interest.

## Footnotes

1 https://phytozome.jgi.doe.gov/pz/portal.html

2 http://www.ncbi.nlm.nih.gov/

3 http://expasy.org/

4 http://www.cbs.dtu.dk/services/SignalP/

5 http://www.cbs.dtu.dk/services/TargetP/

6 http://us.expasy.org/prosite

7 https://www.ebi.ac.uk/interpro/interproscan.html

8 http://blast.ncbi.nlm.nih.gov

9 http://jgi.doe.gov/

10 http://swissmodel.expasy.org/

11 http://bioinfo.ut.ee/primer3-0.4.0/

12 http://engels.genetics.wisc.edu/amplify

## Supplementary material

FIGURE S1 **Predicted 2D (A) and 3D topology (B,C) of GintAMT3**. Models were constructed using Protter – visualize proteoforms (Omasits et al., 2013) and SWISS-MODEL (Benkert et al., 2011). 3D model shows potential tertiary structure of GintAMT3 when incorporated into the membrane **(B)** and from the top from the extracellular side to the intracellular side **(C)**.

FIGURE S2 **Quantification of *GintAMT1* (A) and *GintAMT2* (B) transcripts under phosphate stress**. Gene expression was measured by quantitative polymerase chain reaction in the ERM and ORM of inoculated *P. trichocarpa* and *S. bicolor*. The sorghum and poplar plants grew in a tripartite compartment system where only the fungus had access to the high phosphorus source (open bars) or low phosphorus source (closed bars). Differences between ERM and IRM were tested with a one-way ANOVA. Data were calibrated by the expression values obtained for the gene encoding the transcription elongation factor TEF1α. Values are means of nine replicates, error bars represent SD. Differences between treatments were tested with a one-way ANOVA. Lower case letters indicate significant difference (Tuckey HSD; *p* < 0.05).

TABLE S1 **Primer list**.

TABLE S2 **Mycorrhizal colonization**. Hyphal and arbuscular colonization rates in different treatments were compared by one-way ANOVA. Lowercase letters indicate significant differences between treatments (*p* < 0.05).

## Abbreviations

AM: arbuscular mycorrhiza
AMF: arbuscular mycorrhizal fungi
AMT: ammonium transporter
ERM: extraradical mycelium
HATS: high affinity transport system(s)
IRM: intraradical mycelium
LATS: low affinity transport system(s)
N: nitrogen
GS/GOGAT: glutamine synthetase/glutamate oxoglutarate aminotransferase
