# Supplementary figures and images for: GintAMT3 – a Low-Affinity Ammonium Transporter of the Arbuscular Mycorrhizal Rhizophagus irregularis
Source: Front Plant Sci. 2016 May 25;7:679. doi: 10.3389/fpls.2016.00679 (PMC4879785; doi:10.3389/fpls.2016.00679)

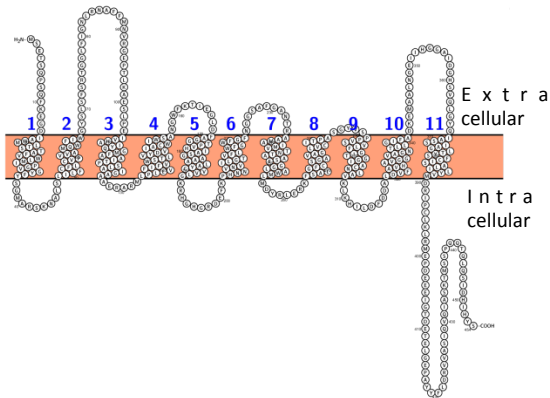

Extra cellular

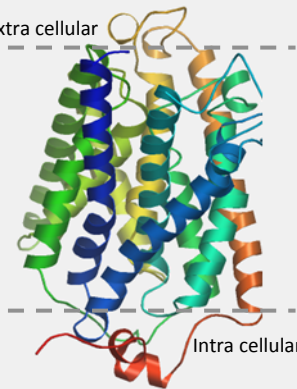

Intra cellular

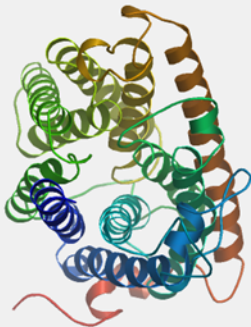

Supplement: FIGURE S1 — Predicted 2D (A) and 3D topology (B,C) of GintAMT3. Models were constructed using Protter – visualize proteoforms (Omasits et al., 2013) and SWISS-MODEL (Benkert et al., 2011). 3D model shows potential tertiary structure of GintAMT3 when incorporated into the membrane (B) and from the top from the extracellular side to the intracellular side (C). [file Image_1.PDF]

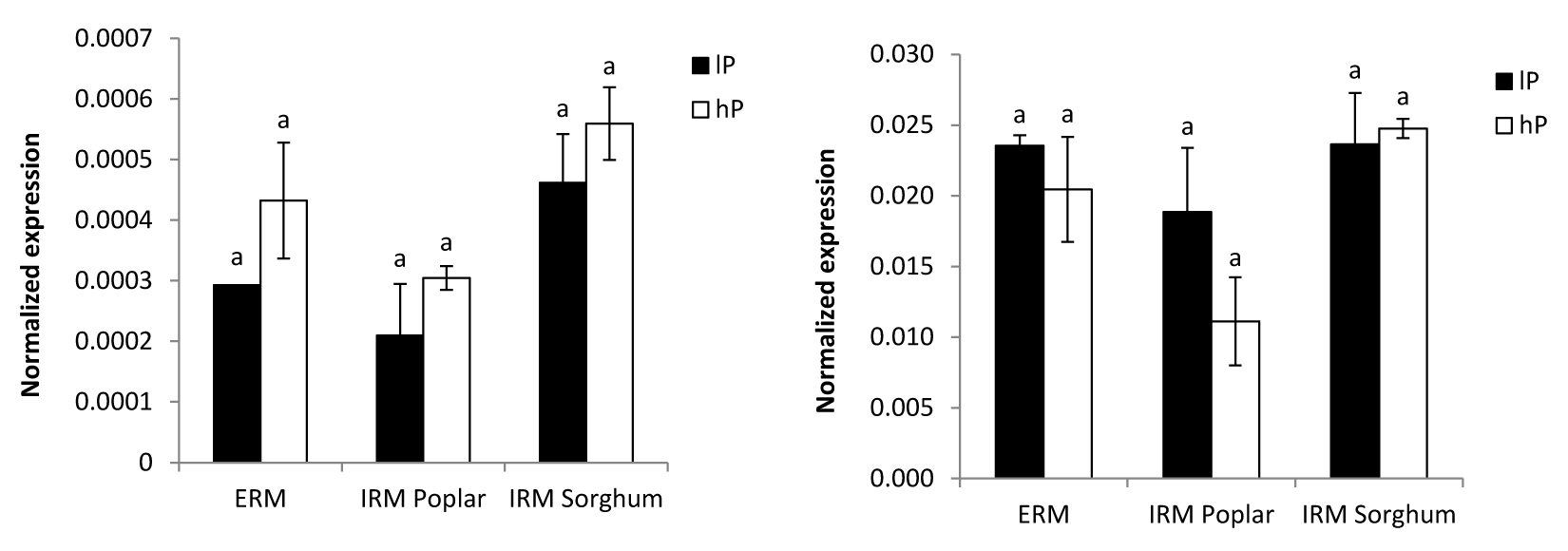

Supplement: FIGURE S2 — Quantification of GintAMT1 (A) and GintAMT2 (B) transcripts under phosphate stress. Gene expression was measured by quantitative polymerase chain reaction in the ERM and ORM of inoculated P. trichocarpa and S. bicolor. The sorghum and poplar plants grew in a tripartite compartment system where only the fungus had access to the high phosphorus source (open bars) or low phosphorus source (closed bars). Differences between ERM and IRM were tested with a one-way ANOVA. Data were calibrated by the expression values obtained for the gene encoding the transcription elongation factor TEF1α. Values are means of nine replicates, error bars represent SD. Differences between treatments were tested with a one-way ANOVA. Lower case letters indicate significant difference (Tuckey HSD; p < 0.05). [file Image_2.TIF]
