# Supplementary material for: GintAMT3 – a Low-Affinity Ammonium Transporter of the Arbuscular Mycorrhizal Rhizophagus irregularis
Source: Front Plant Sci. 2016 May 25;7:679. doi: 10.3389/fpls.2016.00679 (PMC4879785; doi:10.3389/fpls.2016.00679)
Supplement: TABLE S1 — Primer list. [file Table_1.DOCX]

Table S1. Primer list

| **Primer list for Gene amplification** | | |
| --- | --- | --- |
| GintAMT3f | qPCR | GGG CTT GAC TTT GCT GGT |
| GintAMT3r | qPCR | TTC GTC CCT TCC ATG ACC |
| GintAMT3_fl_Fwd | Full length | TTTTCTTTTTCTCCCCCAAGA |
| GintAMT3_fl_Rev | Full length | AAATTAAATAAATGCGAGTGATAGAAA |
| attB1_GintAMT3_fwd | Cloning site | GGGGACAAGTTTGTACAAAAAAGCAGGCTCCTTATGATAAAAAATGTCAG |
| attB2_GintAMT3_rev | Cloning site | GGGGACCACTTTGTACAAGAAAGCTGGGTACACTTTTTTAAGAATAATG |
