# Supplementary material for: GintAMT3 – a Low-Affinity Ammonium Transporter of the Arbuscular Mycorrhizal Rhizophagus irregularis
Source: Front Plant Sci. 2016 May 25;7:679. doi: 10.3389/fpls.2016.00679 (PMC4879785; doi:10.3389/fpls.2016.00679)
Supplement: TABLE S2 — Mycorrhizal colonization. Hyphal and arbuscular colonization rates in different treatments were compared by one-way ANOVA. Lowercase letters indicate significant differences between treatments (p < 0.05). [file Table_2.DOCX]

**Table S2**. Mycorrhizal colonization. Hyphal and arbuscular colonization rates in different treatments were compared by one-way ANOVA. Lowercase letters indicate significant differences between treatments (*p*<0.05).

| **Mean percentage colonization** | | | | | |
| --- | --- | --- | --- | --- | --- |
| **Plant** | **Treatment** | **Hyphae (%)** | | **Arbuscule (%)** | |
|  |  | **Mean** | **SD** | **Mean** | **SD** |
| **Poplar** | **low Pi** | 79.43^a^ | 9.98 | 33.57^a^ | 7.68 |
|  | **high Pi** | 87.29^a^ | 6.29 | 33.57^a^ | 8.48 |
| **Sorghum** | **low Pi** | 93.71^a^ | 5.47 | 15.57^a^ | 6.65 |
|  | **high Pi** | 93.14^a^ | 5.37 | 5.43^b^ | 4.58 |
| **Sorghum** | **N** | 97.00 | 0.82 | 40.25 | 7.89 |
|  | **NO3** | 98.75 | 0.50 | 35.50 | 11.68 |
|  | **NH4** | 94.00 | 0.82 | 46.25 | 2.36 |
